# Pectin-like heteroxylans in the early-diverging charophyte *Klebsormidium fluitans*

**DOI:** 10.1093/aob/mcae154

**Published:** 2024-09-21

**Authors:** Marie N Rapin, John H Bothwell, Stephen C Fry

**Affiliations:** The Edinburgh Cell Wall Group, Institute of Molecular Plant Sciences, The University of Edinburgh, Edinburgh EH9 3BF, UK; Department of Biosciences, Durham University, Durham DH1 3LE, UK; The Edinburgh Cell Wall Group, Institute of Molecular Plant Sciences, The University of Edinburgh, Edinburgh EH9 3BF, UK

**Keywords:** *Klebsormidium*, charophyte, cell wall, algae, polysaccharide, pectin, xylans, rhamnoxylan, galactoxylan, pollution tolerance

## Abstract

**Background and Aims:**

The cell walls of charophytic algae both resemble and differ from those of land plants. Cell walls in early-diverging charophytes (e.g. Klebsormidiophyceae) are particularly distinctive in ways that might enable survival in environments that are incompatible with land-plant polymers. This study therefore investigates the structure of *Klebsormidium* polysaccharides.

**Methods:**

The ‘pectin’ fraction (defined by extractability) of *Klebsormidium fluitans*, solubilized by various buffers from alcohol-insoluble residues, was digested with several treatments that (partly) hydrolyse land-plant cell-wall polysaccharides. Products were analysed by gel-permeation and thin-layer chromatography.

**Key Results:**

The *Klebsormidium* pectic fraction made up ~30–50 % of its alcohol-insoluble residue, was optimally solubilized at pH 3–4 at 100 °C, and contained residues of xylose ≈ galactose > rhamnose > arabinose, fucose, mannose and glucose. Uronic acids were undetectable, and the pectic fraction was more readily solubilized by formate than by oxalate, suggesting a lack of chelation. Some land-plant-targeting hydrolases degraded the *Klebsormidium* pectic fraction: digestion by α-l-arabinanase, endo-β-(1→4)-d-xylanase and α-d-galactosidase suggests the presence of β-(1→4)-xylan with terminal α-l-arabinose, α-d-galactose and (unexpectedly) rhamnose. ‘Driselase’ released oligosaccharides of xylose and rhamnose (~1:1), and graded acid hydrolysis of these oligosaccharides indicated a ‘rhamnoxylan’ with rhamnose side-chains. Partial acid hydrolysis of *Klebsormidium* pectic fraction released rhamnose plus numerous oligosaccharides, one of which comprised xylose and galactose (~1:2 Gal/Xyl), suggesting a galactoxylan. Lichenase was ineffective, as were endo-β-(1→4)-d-galactanase, endo-β-(1→4)-d-mannanase, β-d-xylosidase and β-d-galactosidase.

**Conclusions:**

*Klebsormidium* pectic fraction possesses many land-plant-like linkages but is unusual in lacking uronic acid residues and in containing rhamnoxylan and galactoxylan domains. Uronic acids allow land-plant and late-diverging charophyte pectins to form Ca^2+^-bridges, facilitating cell-wall polymer association; their absence from *Klebsormidium* suggests that neutral heteroxylans rely on alternative cross-linking mechanisms. This lack of dependence on Ca^2+^-bridges might confer on *Klebsormidium* the ability to grow in the acidic, metal-rich environments that it tolerates.

## INTRODUCTION

Charophytic green algae (charophytes) are the closest living relatives to land plants, and the two groups share many evolutionary adaptations. Cell walls are one of these shared adaptations, but the charophyte lineage is a large and diverse one (some species are aquatic and others terrestrial, and multiple morphologies and sizes are found across the clade), hence charophyte cell-wall structures and polymers are correspondingly diverse. Together with land plants, the ‘late-diverging’ charophyte classes (Zygnematophyceae, Coleochaetophyceae and Charophyceae) comprise the Phragmoplastophyta (*sensu*Hall *et al.*, 2020) and, as in land plants, the extractable pectic fractions of the cell walls of the late-diverging charophytes are rich in homogalacturonan with varying degrees of methyl-esterification (Cherno *et al.*, 1976; Popper and Fry, 2003; Domozych *et al.*, 2007, 2009, 2010; Eder and Lütz-Meindl, 2008, 2010; Sørensen *et al.*, 2011; O’Rourke *et al.*, 2015; Anderson, 2016). Land-plant-like xyloglucan has also been identified chemically in a few late-diverging charophyte species (Mikkelsen *et al.*, 2021). In contrast, the cell walls of the more basal, ‘early-diverging’ charophyte classes (Klebsormidiophyceae, Chlorokybophyceae and Mesostigmatophyceae — Cracraft and Donoghue, 2004; Cook and Graham, 2017) have been less well studied.

Of these early-diverging classes, the Klebsormidiophyceae hold particular interest for studies of cell-wall evolution. The class has been the subject of much taxonomic discussion because its members are often hard to assign (Glass *et al.*, 2023): they all form unbranched uniseriate filaments that display highly variable and environmentally sensitive morphologies, and they often clump into thick, deep-green mats. Strikingly, however, species in the genus *Klebsormidium* can live in highly diverse environments, from damp, shaded sites in European cities to more extreme locations, such as alpine glaciers and soil crusts (Karsten *et al.*, 2010, 2013; Karsten and Holzinger, 2014; Kitzing *et al.*, 2014), arid and sandy South African deserts (Karsten *et al.*, 2016), polar regions (Elster *et al.*, 2008; Rippin *et al.*, 2019) and acid mine drainages (Škaloud *et al.*, 2014). This range of habitats is apparently enabled by a similar range of physiological adaptations (Holzinger and Pichrtová, 2016). Some *Klebsormidium* species, for example, produce specific metabolites that protect against desiccation (Kaplan *et al.*, 2012), excessive ultraviolet irradiation (Karsten and Holzinger, 2014), cold (Nagao *et al.*, 2008) or multiple environmental stresses (Míguez *et al.*, 2020).

In addition to these metabolic adaptations and/or acclimations, there is increasing evidence that cell-wall adaptations also contribute to the success of the genus *Klebsormidium*. In response to desiccation stress, certain species stain positively for callose in their cell walls (Herburger and Holzinger, 2015) and, more enigmatically, *Klebsormidium* cell walls display unusual and distinctive compositions (Rapin *et al.*, 2023). Most notably, biochemical and immunological studies (Sørensen *et al.*, 2011; O’Rourke *et al.*, 2015) have shown that galacturonic acid, which is a major chelating component of land-plant pectin, is missing from the *Klebsormidium* cell wall. In most land plants, these uronic acid-containing pectic domains are essential for cell–cell adhesion and cell shaping (Jarvis *et al.*, 2003), cell immunity (Wang *et al.*, 2023) and signalling (Albersheim *et al.*, 2011).

Accordingly, we report here an improved characterization of *Klebsormidium* ‘pectin’ (*sensu lato*), defined here operationally as the polysaccharide fraction solubilized by standard treatments for the extraction of classic pectin in land plants and late-diverging charophytes. Our findings demonstrate a number of unusual polymer structures that might contribute to the wide environmental tolerance of *Klebsormidium* species.

## MATERIALS AND METHODS

### Materials

*Klebsormidium fluitans* was purchased from the Culture Collection of Algae and Protozoa, Scottish Marine Institute, Dunstaffnage, Argyll, UK (https://www.ccap.ac.uk/). Algae were grown on Bold basal medium (3N-BBM + V) (Bischoff and Bold, 1963) as the only photosynthetic organism present.

Driselase and all chemicals and solvents were obtained from Sigma-Aldrich (https://www.sigmaaldrich.com). All other enzymes were sourced from Megazyme (https://www.megazyme.com). Thin-layer chromatography (TLC) plates were Merck silica-gel 60 plastic-backed plates (https://www.merckgroup.com), and beads for the gel-permeation chromatography were from Bio-Rad (https://www.bio-rad.com). Data analysis was performed in R (R Core Team, 2021).

### Alcohol-insoluble residue pretreatments

Freeze-dried algal biomass was stirred for 16 h with 70 % ethanol, centrifuged at 5000*g* for 10 min, and the supernatant was discarded. The process was repeated three to five times with 70 % ethanol, finishing with final washes of 96 % ethanol, then acetone. The washed cells were air-dried at room temperature to leave alcohol-insoluble residue (AIR; all abbreviations are listed in the Appendix).

Pretreatment A (alkali digestion) was performed by incubation of AIR at 5 mg mL^–1^ in 1 M NaOH in 75 % ethanol at 20 °C for 10 min. The mixture was centrifuged at 4000*g* for 10 min, then washed sequentially in 75 % ethanol, 100 % ethanol and ethanol containing 0.5 % acetic acid (10–20 min each).

Pretreatment E [endopolygalacturonase (EPG) digestion] was performed by incubation of AIR (20 mg mL^–1^) in 2.5 U mL^–1^ EPG in 160 mM acetate (pyridine^+^, pH 4.7) at 25 °C for 16 h, and the residue was rinsed with water twice. [Note: all buffer concentrations are given as the sum of the charged + uncharged form(s).] Sequential application of pretreatments A and E is referred to as A+E.

Pretreatment αA (α-amylase digestion) was performed after pre-incubation of AIR (10 mg mL^–1^) at 100 °C in aqueous 40 mM lutidine buffer (acetate^−^, pH 6.7) containing 0.25 % w/w chlorobutanol (antimicrobial) for 15 min, followed by cooling to 60 °C and incubation for 72 h with 4 U mL^–1^ heat-resistant α-amylase (prepared from 10 mL of commercial solution dialysed against water and diluted 4.5-fold in the lutidine buffer). The mixture was then supplemented with ethanol and ammonium formate (to 70 % v/v and 1 % w/v, respectively) and incubated at 20 °C for 16 h, thus coagulating any polymers that had been dissolved by the heat treatments but not digested; the total insoluble material was washed in 70 % ethanol several times.

After each pretreatment, the remaining AIR was rinsed with acetone and dried in a fume hood.

### Sequential extraction of polysaccharides from AIR

After these pretreatments, AIR was submitted to a sequential extraction process, starting with 0.2 M oxalate buffer (ammonium, pH 4.1) at 100 °C for 2 h. After centrifugation, the supernatant was taken as pectic fraction P1. The remaining pellet was incubated in identical conditions for an extra 16 h, and the supernatant was taken as P2. P1 and P2 were dialysed against water. All dialysis (unless otherwise stated) was performed in 12-kDa molecular-weight cut-off tubing. The pellet remaining after both hot oxalate treatments was incubated in 6 M NaOH for 72 h at 37 °C, i.e. optimal conditions for extracting land-plant hemicelluloses (Edelmann and Fry, 1992). After centrifugation, the supernatant (‘hemicellulose’) was acidified to around pH 4.7 with acetic acid, dialysed against water and centrifuged, giving a pellet (hemicellulose a fraction; Ha) and supernatant (Hb). The AIR pellet remaining after NaOH treatment was washed with 0.2 M acetate buffer (Na^+^, pH 4.7), and the supernatant was collected by centrifugation (‘wash’ fraction; W) and dialysed against water. The final insoluble residue was rinsed thoroughly with water and taken as α-cellulose (αC). All centrifugation steps were performed at 5000 *g* for 10 min. All six fractions were freeze-dried after dialysis against water (P1, P2, Hb and W) or rinsing in water (Hb and αC).

### AIR extractability assays

In preliminary work, we monitored the extractability of the operationally defined pectic fractions P1 and P2 in various conditions, including three buffers: 0.2 M oxalate, acetate or formate (ammonium, pH 4.1) at 20, 60 and 100 °C. Similar buffers were also tested at pH 3.0–7.0 and 100 °C for 2 and 16 h. The pectin fractions thus extracted were dialysed against water (in 4-kDa molecular weight cut-off tubing) and freeze-dried.

### Acid hydrolysis

For complete acid hydrolysis of non-cellulosic poly- or oligosaccharides, samples were dissolved or suspended at 5 mg mL^–1^ in 2 M trifluoroacetic acid (TFA) and incubated at 120 °C for 1 h. They were then dried, re-dissolved in water, re-dried and, finally, diluted to the desired concentration. For partial acid hydrolysis, samples were dissolved or suspended at 5 mg mL^–1^ in 0.5 M TFA and incubated at 80 °C for 1 h, then processed as above.

### Enzymic digestion

Enzymic digestions were performed as in Table 1. After incubation, ethanol was added to 75 % v/v, samples were incubated on a wheel at ~20 °C for ≥6 h, then centrifuged at 3000*g* for 10 min; 90 % of the supernatant was recovered, dried in a SpeedVac and, finally, re-dissolved in water to the appropriate concentration.

**Table 1.** Enzymes and conditions used to dissect polysaccharides^a^

Enzyme features			Incubation				
Enzyme	Source	Specific activity (U mg^−1^)	Enzyme concentration (U mL^–1^)	Buffer	Substrate concentration (mg mL^–1^)^b^	Time (h)	Temperature (°C)
Endo-β-(1→4)-d-galactanase	*Aspergillus niger*	>150	1.25	85 mM acetate (pyridine^+^, pH 4.0)	1.25	1	25
Endo-α-(1→5)-l-arabinanase	*Aspergillus niger*	~10	2.5	85 mM acetate (pyridine^+^, pH 4.0)	1.25	1	20
Endo-β-(1→4)-d-mannanase (mannanase 1)	*Bacillus* sp.	>50	5	25 mM ammonium (acetate^−^, pH 8.8)	1.25	1	20
Endo-β-(1→4)-d-mannanase (mannanase 2)	*Aspergillus niger*	~50	5	250 mM pyridine (formate^−^, pH 3.7)	1.25	1	20
Endo-β-(1→4)-d-xylanase	Rumen microorganisms	~ 380	5	40 mM lutidine (acetate^−^, pH 6.5)	1.25	16	20
Lichenase	*Bacillus subtilis*	~230	5	40 mM lutidine (acetate^−^, pH 6.5)	1.25	1	40
β-d-Xylosidase	*Selenomonas ruminantium*	~115	8.33	8.33 mM succinate (Na^+^, pH 5.5)	8.33	0.5	40
β-d-Galactosidase	*Aspergillus niger*	~170	0.33	56.67 mM acetate (pyridine^+^, pH 5)	6.67	48	25
α-d-Galactosidase	Guar	~50	50	85 mM acetate (pyridine^+^, pH 5)	1.25	48	25
Driselase	*Irpex lacteus*	N/A	0.02 %	120 mM pyridine (acetate^−^, pH 4.7)	9.62	16–90	37

^a^The enzyme was dissolved in buffer, then mixed with an aqueous suspension or solution of the substrate(s) to give the final concentrations indicated (total volume 20–31 µL).

^b^Substrates were dissolved or suspended in water with 0.5 % chlorobutanol prior to incubation. All enzymes were applied to the *Klebsormidium* pectic fraction. Additionally, galactanase was assayed on commercial β-(1→4)-galactan; arabinanase was assayed on commercial α-(1→5)-arabinan; lichenase was assayed on lichenan, barley mixed-linkage (1→3),(1→4)-β-d-glucan (high and medium viscosity), starch and cellulose; and β- and α-galactosidase were tested on commercial β-(1→4)-galactan.

### Thin-layer chromatography

Analytical TLC was performed on plastic-backed silica-gel plates unless indicated otherwise, mostly in butan-1-ol/acetic acid/water [4:1:1, v/v/v (BAW)] or ethyl acetate/pyridine/acetic acid/water [6:3:1:1, v/v/v (EPAW)]. Sugars were stained with thymol/H_2_SO_4_ (Jork *et al.*, 1994).

Preparative TLC was performed on glass-backed silica-gel plates in the above solvents. The silica layer was cut into strips, and each strip was eluted into a small volume of water. The suspension was incubated under agitation for ≥16 h, then centrifuged (4000*g*, 5 min) and the supernatant collected. The process was repeated three times. The eluates were combined, dried and redissolved in water at 5 mg mL^–1^.

### Sugar quantification

Sugar quantification was performed on pieces of plastic-backed silica-gel plate. Standard solutions of Glc (0.125–5.0 mg mL^–1^) in addition to the samples of interest were dried on a plate as 1–3-µL dots, then (without chromatography) stained with thymol/H_2_SO_4_ (Jork *et al.*, 1994). The intensity of the staining was measured with ImageJ and MatLab, referenced to the Glc dilution series, and the concentrations in the samples of interest were calculated (Glc equivalents). Each sample of interest was assayed on three replicate pieces of TLC plate, and data were averaged ± s.d. (Rapin *et al.*, 2023).

### Gel-permeation chromatography

Gel-permeation chromatography was performed on a Bio-Gel P-30 column (160-mL bed volume) in 160 mM acetate (pyridine^+^; pH ~4.7). Samples of ≤5 mL were loaded, and 2-mL fractions were collected. The column was routinely flushed for several hours between runs. Gel-permeation chromatography on a Bio-Gel P-2 column (90-mL bed volume) was performed in an identical fashion.

## RESULTS

### *Extractability of a* Klebsormidium *pectic fraction: impact of chemical and enzymic pretreatments*

**Fig. 1.**
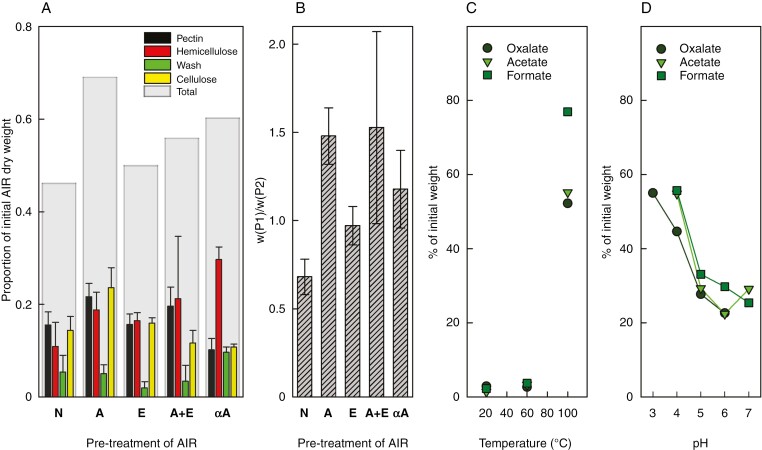
*Klebsormidium* polysaccharide fractions and their extractability. (A) Proportions of the sequentially extracted polysaccharide fractions (dialysed in 12-kDa molecular weight cut-off tubing) following a variety of pretreatments, as proportion of the initial untreated alcohol-insoluble residue (AIR) weight; ‘pectin’ = pectic fractions P1 + P2; ‘hemicellulose’ = Ha + Hb; ‘wash’ = W; ‘cellulose’ = α-cellulose. Pretreatments were as follows: N, none; A, ethanolic alkali; E, endo-α-(1→4)-poly-d-galacturonase; A+E, A and E applied sequentially; αA, α-amylase. Error bars indicate standard deviations (*n* = 3). (B) Ease of pectic fraction extractability quantified as the P1/P2 weight ratio observed after each pretreatment. Error bars indicate standard deviations (*n* = 3). (c) Effect of temperature and buffer salt on pectic fraction extraction from *Klebsormidium* AIR that had not been pretreated [i.e. ‘N’ of (A)]. The oxalate, acetate and formate buffers were all 200 mM [sum of acid + anion(s)], pH 4.1, and with NH_4_^+^ as the counter-ion. The extracted pectic fraction was assayed by weight, after dialysis against water [in 4-kDa molecular weight cut-off tubing; cf. 12-kDa tubing in (A) and (B)]. (D) Influence of pH on pectic fraction extractability in the same three 200 mM buffers, at 100 °C, and with NH_4_^+^ as a counter-ion.

Extraction of an operationally defined pectic fraction was performed on *Klebsormidium* alcohol-insoluble residue (AIR, i.e. essentially total cellular polymers, hence enriched in cell-wall polysaccharides). We describe the polymer fractions thus extracted as ‘pectin’, regardless of chemical composition. Extractability of the major cell-wall fractions was tested in *Klebsormidium* AIR that had been subjected (or not) to a range of enzymic and chemical pretreatments (Fig. 1A). The extractants used after these pretreatments were based on those conventionally applied to land-plant cell walls: extraction of ‘pectins’ P1 and P2 in boiling oxalate at pH 4.1, ‘hemicelluloses’ Ha and Hb in 6 M NaOH at 37 °C, and a pH 4.7 buffer wash (W), leaving unextracted cellulose (αC).

The total yields of fractionated polymers were between ~50 and 70 % of the initial AIR weight; the partial losses could be linked to the dialysis process in 12-kDa molecular weight cut-off tubing, which might have permitted some smaller polymers to escape. The cell wall was initially fractionated without any pretreatment (N in Fig. 1A): roughly one-third of the extracted polymers were ‘pectic’, another one-third were hemicelluloses plus the wash fraction, and the remaining one-third was counted as α-cellulose. ANOVA suggested that our different extraction conditions (A, E, A+E and αA in Fig. 1A) did not affect hemicellulose yields [*F*(4,10) = 2.2, *P* = 0.15], but did significantly affect the relative pectic fraction yields [*F*(4,10) = 8.8, *P* < 0.01], as discussed in the next three paragraphs.

Land-plant pectin contains homogalacturonan that is partly methyl-esterified and thus not fully susceptible to EPG digestion. To check for the presence of any homogalacturonan in *Klebsormidium*, we de-esterified the AIR with alkali, then incubated it with EPG (A+E in Fig. 1A); controls were treated with alkali only or EPG only (A and E, respectively, in Fig. 1A). Tukey’s *post hoc* test showed that none of these three pretreatments decreased the relative yield of the pectic fraction compared with the untreated AIR (N), suggesting the absence of homogalacturonan in *Klebsormidium*.

**Fig. 2.**
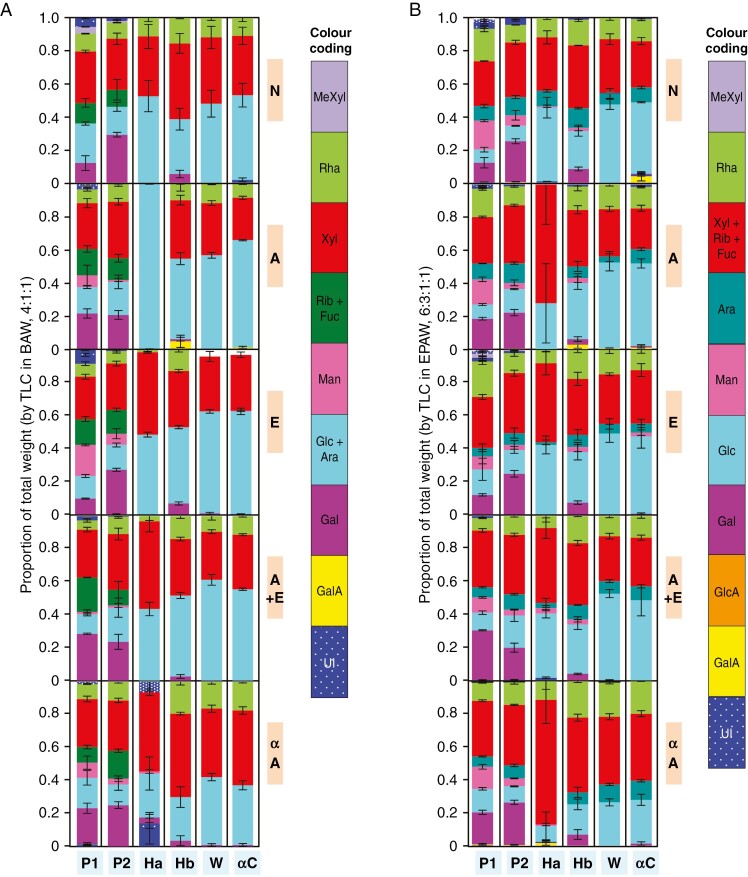
Relative quantities of sugar residues in *Klebsormidium* polysaccharide fractions extracted from alcohol-insoluble residue (AIR) following various pretreatments. AIR was pretreated, or not, as described in Fig. 1A (N, A, E, A+E and αA), then the six polymer fractions were separated (P1, P2, Ha, Hb, W and αC). Each fraction was acid-hydrolysed (2 M TFA, 120 °C, 1 h), and the resulting monosaccharides were analysed by TLC in (A) BAW, two ascents, or (B) EPAW, two ascents. The error bars indicate the s.d. of the quantity of each sugar (*n* = 3, *n* being the number of replicates of each polysaccharide fraction from the same AIR sample). UI, minor sugar(s) that could not be identified with any tested markers. Scans of the TLCs were processed with ImageJ and MatLab to quantify the sugars, as described by Rapin *et al.* (2023).

Alternatively to these homogalacturonan-targeting pretreatments, the *Klebsormidium* AIR was pretreated with thermostable α-amylase (αA; aimed at solubilizing starch) prior to sequential polymer extraction, because Glc, the product of acid hydrolysis of starch, might confuse cell wall analyses. Tukey’s *post hoc* test suggested that the αA pretreatment caused the proportion of the pectic fraction to drop significantly (*P* < 0.05) to about half of the values seen in the A and A+E treatments, with yields from the N and E treatments lying roughly in the middle. We interpret these results as indicating the solubilization of some alkaline-extractable non-homogalacturonan ‘pectin’ at the same time as starch, thus diminishing the measurable pectic fraction (cf. Fig. 2).

The pectic fraction was extracted in two parts (P1 and P2) by hot oxalate sequentially for 2 and 16 h, respectively. To evaluate the extractability of the pectic fraction, we report the weight ratio of P1/P2 (Fig. 1B). In AIR that had received no pretreatment (N), P1/P2 was ~0.6, showing that about one-third of the extractable pectic fraction had been extracted during the first 2 h. ANOVA suggested that different extraction conditions had a significant effect on P1/P2 ratios [*F*(4,10) = 4.7, *P* < 0.02], with Tukey’s *post hoc* test suggesting that A and A+E pretreatments significantly enhanced extractability compared with the N treatment (*P* < 0.05), supporting the idea that pre-incubating *Klebsormidium* AIR in 1 M NaOH at room temperature promoted the extractability of cell-wall polymers.

### *Extractability of* Klebsormidium *pectic fraction: impact of extraction process*

Factors affecting the extractability of the pectic fraction from untreated algal AIR (i.e. sample N in Fig. 1A, B) were evaluated with a range of pH and temperature conditions (Fig. 1C, D). At low temperatures (20 and 60 °C), very little polysaccharide was extracted within 2–16 h in any of three pH 4.1 buffers (oxalate, acetate and formate). In comparison, at 100 °C, 50–80 % of the initial AIR mass was extracted as P1 + P2, and the different buffers appeared to differ in effectiveness (formate > acetate ≈ oxalate) (Fig. 1C).

The impact of pH on the extractability of the pectic fraction was measured at 100 °C for each buffer (Fig. 1D). In most cases, lowering the pH promoted pectic fraction extraction. A possible exception to this trend was the increase seen in acetate between pH 6.0 and 7.0. The same order of effectiveness was noted as in Fig. 1C (formate > acetate ≈ oxalate), although the trend was weaker in this experiment.

### Sugar residue composition of extracted polysaccharide fractions

The pectic fraction extracted by various pH 4.1 buffers and temperatures (as in Fig. 1C) exhibited few differences in monosaccharide residue profile (Supplementary Data Fig. S1), showing that essentially the same polysaccharide(s) had been extracted regardless of the extraction temperature (20, 60 or 100 °C) and time (2 vs. 16 h) and the nature of the buffer. The sugar composition data are summarized in Table 2; details are as follows.

**Table 2.** Acid- and enzyme-catalysed hydrolysis of *Klebsormidium* pectic fraction extract (P2).

Catalyst	Target polysaccharides of land plants	Product(s) from *Klebsormidium* pectic fraction (and conclusions drawn)	See Fig.
Severe acid (2.0 M TFA, 120 °C, 1 h)	All glycosidic linkages except in cellulose	Xyl ≈ Gal > Rha > Ara, Fuc, Man, Glc	2
Moderate acid (0.5 M TFA, 80 °C, 1 h)	Relatively acid-labile linkages (especially furanose residues) in non-cellulosic polysaccharides	Monosaccharides [Ara_(furanose)_ > Rha > Gal > Xyl, Fuc, Man, Glc]; oligosaccharides rich in Gal + Xyl	8, 9
Mild acid (0.1 M TFA, 80 °C, 0–16 h)	Relatively acid-labile linkages in non-cellulosic polysaccharides	First Rha; later Xyl (thus the Rha was mainly present as side-chains)	7
Endo-β-(1→4)-d-galactanase	Galactan and type I arabinogalactans (rhamnogalacturonan-I side-chain, pectin)	None (thus no mid-chain β-d-Gal)	3
Endo-α-(1→5)-l-arabinanase	Arabinan (rhamnogalacturonan-I side-chain, pectin)	Ara (thus α-l-linked), Prod2	3
Endo-β-(1→4)-d-xylanase	Xylan, arabinoxylan and methylglucuronoxylan (hemicellulose)	Prod3, Prod4 (thus containing mid-chain β-d-Xyl)	4
Endo-β-(1→4)-d-mannanase 1	Mannan, glucomannan (hemicellulose)	None (thus no mid-chain β-d-Man)	4
Endo-β-(1→4)-d-mannanase 2	Mannan, glucomannan (hemicellulose). Side action on arabinoxylan and methylglucuronoxylan (hemicelluloses)	Prod4 (mannanase 2 possesses some xylanase activity)	4
Lichenase	Mixed-linkage glucan (hemicellulose)	None (thus no mixed-linkage (1→3),(1→4)-β-d-glucan)	4
β-d-Xylosidase	Terminal Xyl residues of (hetero)xylans and xylogalacturonan	None (thus no terminal β-d-Xyl residues)	5
α-d-Galactosidase	Galactoglucomannans	Gal (thus α-linked, unlike in land-plant rhamnogalacturonan-I), ?Rha	5
β-d-Galactosidase	Terminal Gal residues of rhamnogalacturonan-I etc.	None (thus no terminal β-d-Gal residues)	5
Driselase	All except rhamnogalacturonan-II and, possibly, arabinogalactan-proteins	Xyl (thus β-linked, unlike in land-plant xyloglucan), Ara, Glc, oligomer series	6

The sugar residue compositions of the six polysaccharide fractions extracted after each of the five AIR pretreatments are shown in Fig. 2. The most striking result is the virtual absence of uronic acid residues in all fractions. In land plants and in late-diverging charophytes, galacturonate would represent a major proportion of the cell-wall sugar residues in pectins P1 and P2. In the *Klebsormidium* pectic fraction, in contrast, only neutral sugars were detected, in order of abundance: xylose (Xyl) > galactose (Gal) > glucose (Glc) > arabinose (Ara) > rhamnose (Rha) > mannose. In untreated and EPG-treated AIR samples (N and E), Gal was noticeably less abundant in P1 than in P2. This corresponds to the lowest P1/P2 ratios (Fig. 1B). Moreover, the Glc residues did not diminish after de-starching (N compared with αA), suggesting that most of the Glc is an integral part of the *Klebsormidium* pectic fraction rather than of starch. Finally, in almost all extracts, small quantities of fast-migrating compounds (probably *O*-methylated and/or deoxy sugars) were observed. All the non-‘pectic’ polysaccharide fractions (Ha, Hb, W and αC) were similar to each other in sugar residue composition, with Glc and Xyl as their main components and with smaller amounts of Rha and Ara. The presence of Rha, Xyl and Ara in the ‘αC’ fraction suggests that the polymeric structure(s) containing these sugars have a strong affinity for cellulose.

### *Enzymic dissection of* Klebsormidium *pectic fraction*

Given the unique composition of the *Klebsormidium* pectic fraction, we explored the glycosidic linkages of fraction P2 (chosen over P1 because of its higher yield) by dissection with a series of endo- and exo-hydrolases to characterize its sugar residues and their linkages. The results are summarized in Table 2; details are as follows.

**Fig. 3.**
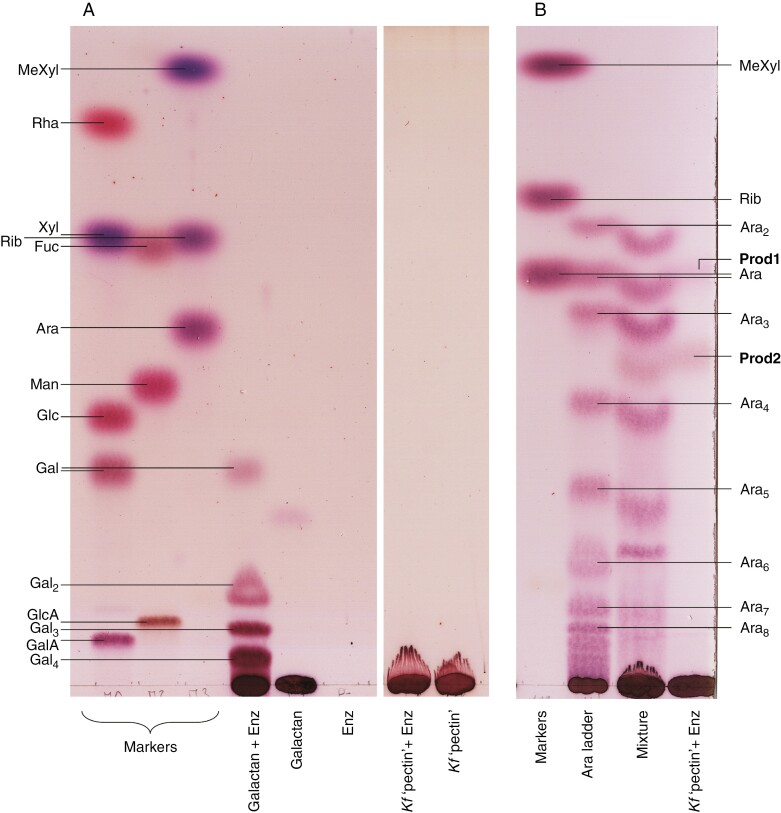
Enzymic digestion of *Klebsormidium* pectic fraction with enzymes that target land-plant pectin. (A) Endo-β-d-galactanase digestion: commercial β-(1→4)-galactan and *Klebsormidium* pectic fraction were incubated with or without endo-β-(1→4)-d-galactanase (Enz); the enzyme was also incubated with no substrate as a control. The markers are monosaccharides; putative galacto-oligomers (Gal_2_ to Gal_4_) are also labelled. Note: two sections of the same plate were attached together digitally for clarity. (B) α-l-Arabinanase digestion: *Klebsormidium* pectic fraction was incubated with endo-α-(1→5)-l-arabinanase (Enz), and commercial α-(1→5)-arabinan was acid-hydrolysed to produce an oligosaccharide ladder. These two samples were chromatographed alone and as a mixture, potentially revealing co-migration of products. Both TLCs were run in EPAW with two ascents. When enzyme or polysaccharide was omitted, the buffer and incubation time were the same as without the omission.

Two hydrolases that digest land-plant pectins were tested: endo-β-(1→4)-d-galactanase and endo-α-(1→5)-l-arabinanase (Fig. 3), both of which attack the side-chains of land-plant rhamnogalacturonan-I. The galactanase digested potato β-(1→4)-galactan, but not *Klebsormidium* P2 (Fig. 3A). In contrast, the arabinanase released two compounds from P2: one identified as Ara by co-chromatography (‘Prod1’ in Fig. 3B), and a second, slower-migrating one, ‘Prod2’. The latter did not fit the ladder pattern of acid-hydrolysed α-(1→5)-arabinan, suggesting that the oligomer contained different sugars (such as Gal), was not α-(1→5)-linked and/or was not linear. [Note that α-(1→5)-Ara_2_ is unusual in migrating faster than the corresponding monosaccharide in the EPAW TLC system (Nguyen-Phan and Fry, 2019) and in some others (Margolles and de los Reyes-Gavilán, 2003).]

**Fig. 4.**
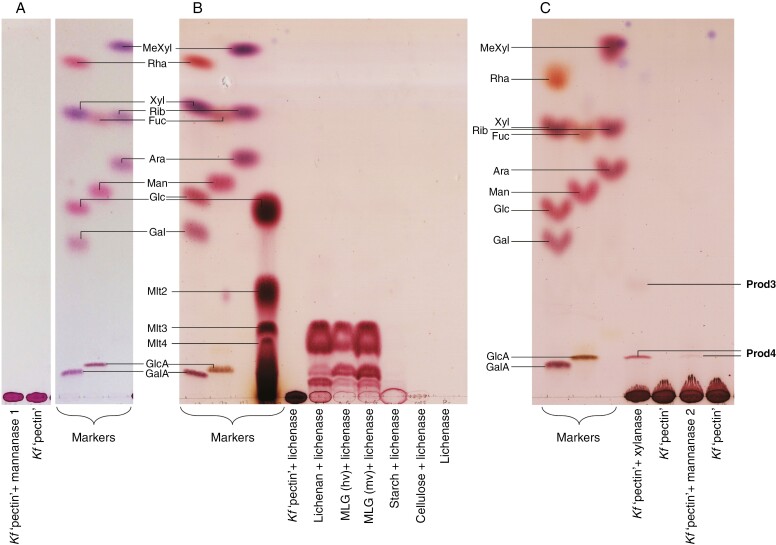
Enzymic digestion of *Klebsormidium* pectic fraction by hemicellulose-active enzymes. (A) Effect of endo-β-(1→4)-d-mannanase 1. The pectic fraction was incubated with or without mannanase 1. Note: two sections of the same plate were attached together digitally for clarity. (B) Effect of lichenase. The pectic fraction, various glucans and a substrate-free control were incubated with lichenase. (C) Effect of endo-β-(1→4)-d-mannanase 2 or endo-β-(1→4)-d-xylanase. The pectic fraction was incubated with xylanase, mannanase 2 or their respective buffers (labelled "*Kf* 'pectin'"). In all cases, analysis was by TLC in EPAW with two ascents, and monosaccharides were used as markers; (B) also includes a malto-oligosaccharide ladder (from Mlt_2_ to Mlt_4_). When enzyme or polysaccharide was omitted, the buffer and incubation time were the same as without the omission.

Next, four hydrolases that attack land-plant hemicelluloses were tested on the *Klebsormidium* pectic fraction. Neither lichenase [active on mixed-linkage (1→3),(1→4)-β-d-glucan (MLG)] nor endo-β-(1→4)-d-mannanase 1 [active on β-(1→4)-mannan and -glucomannan] released any sugar from *Klebsormidium* P2 (Fig. 4A, B), whereas both were active on their specific substrates, MLG (Fig. 4B) and mannan (not shown), respectively. Lichenase was also tested on cellulose and starch, and in buffer alone, demonstrating the selectivity of the enzyme for hydrolysis of MLG (Fig. 4B). Finally, endo-β-(1→4)-d-xylanase (active on arabinoxylan and 4-*O*-methylglucuronoxylan) and endo-β-(1→4)-d-mannanase 2 (active on mannan and glucomannan) both released oligomeric compounds Prod3 and Prod4 (Fig. 4C). Although Prod4 co-migrated with glucuronate, it was not a common uronic acid, as shown by its colour of staining: purple instead of brown.

**Fig. 5.**
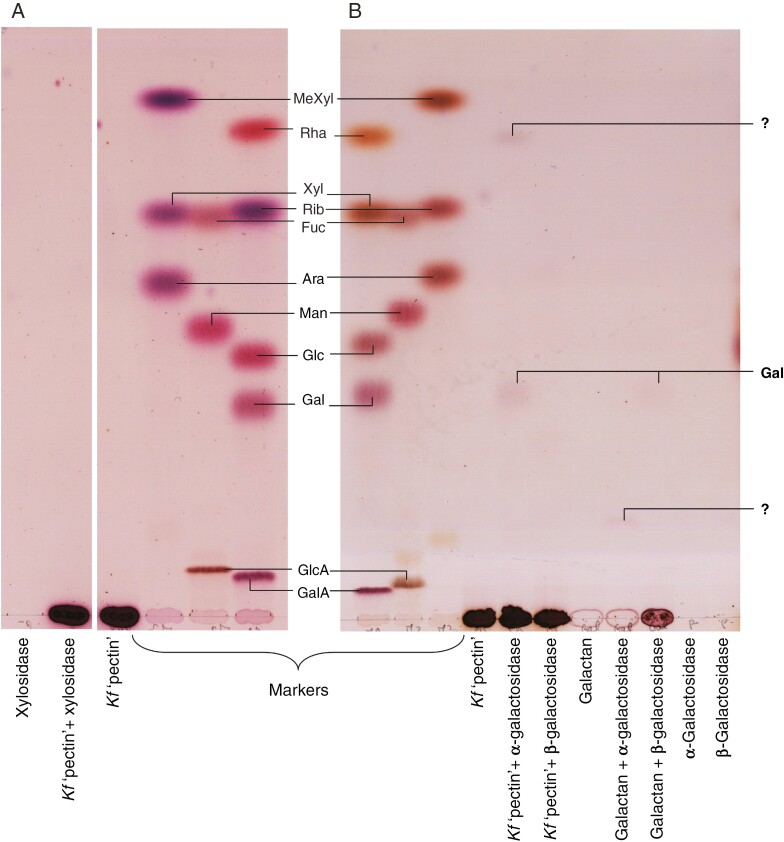
Enzymic digestion of *Klebsormidium* pectic fraction with exo-enzymes. (A) The pectic fraction or a substrate-free control was incubated with or without β-d-xylosidase. (B) The pectic fraction or commercial β-(1→4)-galactan was incubated with α- or β-d-galactosidase. Controls lacked either enzyme or polysaccharide. In all cases, analysis was by TLC in EPAW with two ascents. When enzyme or polysaccharide was omitted, the buffer and incubation time were the same as without the omission.

In addition, a range of exo-hydrolases was trialled on the *Klebsormidium* pectic fraction. β-d-Xylosidase had no effect (Fig. 5A). d-Galactosidases (α- and β-) were tested on β-(1→4)-galactan, and Gal was released only by the β-galactosidase, as expected (Fig. 5B). However, when incubated with the *Klebsormidium* pectic fraction, only the α-galactosidase released any Gal. Another (unidentified) compound, co-migrating with Rha, was also released. Its staining colour was difficult to determine owing to its low abundance, and it could not be identified reliably as Rha. All samples loaded were ethanolic supernatants obtained from the incubated samples; ‘pectin’-containing samples yielded oligomers of intermediate size, soluble in ethanol but immobile on TLC (heavy band at the origin in Fig. 5A, B). Galactan-containing samples showed no such oligomers (faint bands at the origin in Fig. 5B).

**Fig. 6.**
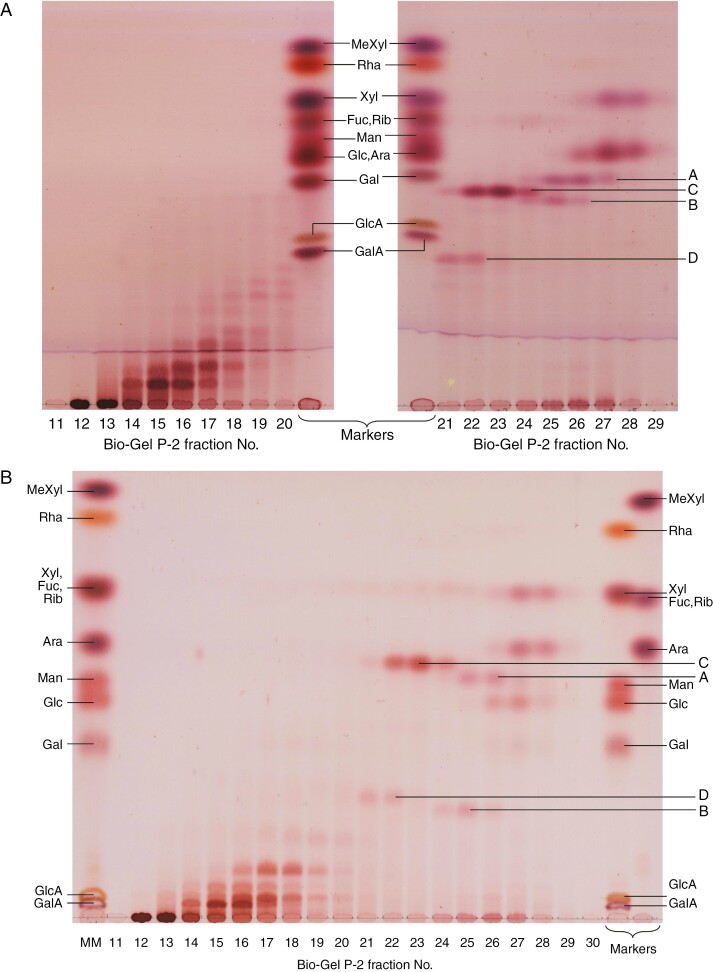
Driselase-digestion products of *Klebsormidium* pectic fraction after pretreatment with amylase. *Klebsormidium* pectic fraction P2 from non-pretreated alcohol-insoluble residue (type ‘N’ of Fig. 1A) was digested with α-amylase, and the resultant ethanol-insoluble material (including that which had dissolved in the aqueous amylase solution) was digested with Driselase. The new ethanol-soluble fraction was dried, re-dissolved in water and size-fractionated on a 90-mL bed-volume column of Bio-Gel P-2. Two-millilitre fractions were collected, and the thymol-positive ones were run by TLC in BAW (A) or EPAW (B).

Finally, we tested the digestion of α-amylase-pretreated *Klebsormidium* pectic fraction P2 with Driselase, which is a commercial mixture of fungal enzymes that hydrolyse most of the polysaccharides of land-plant cell walls to yield mono- and disaccharides (Fry, 2000). The ethanol-soluble products were analysed by gel-permeation chromatography on Bio-Gel P-2, which revealed oligomers with degrees of polymerization (DP) from 1 to 9 (Fig. 6). Free monosaccharides (Xyl, Ara and Glc) were detectable (peaking in fractions 27–28), indicating that these monosaccharides had been linked within the pectic fraction by Driselase-labile bonds. Short oligomers (A–D) were visible in fractions 21–27; these migrated at very different speeds in the two TLC solvent systems used, and all stained pink (indicating hexose residues, such as Glc), with the exception of oligomer A, which stained a darker purple (indicating pentoses, such as Xyl). Judged by retention time on Bio-Gel P-2, A and B were most likely to be disaccharides, C a trisaccharide and D a tetrasaccharide. Earlier Bio-Gel fractions (14–20) contained various larger oligomers, and fractions 12 and 13 contained oligomers that were soluble in ethanol but immobile on TLC, hence larger still.

The larger TLC-mobile Driselase-released oligosaccharides (fractions 14–20 in Fig. 6) were separated by preparative TLC with three ascents in EPAW. Ten sub-fractions were obtained, which were re-grouped by similarity, affording ten oligomers (D1–D10) that were separable by TLC in BAW (Supplementary Data Fig. S2A). Upon total acid hydrolysis, each of these released mainly Rha and Xyl (Supplementary Data Fig. S2B); other minor sugars were also detectable, such as Ara and Glc.

**Fig. 7.**
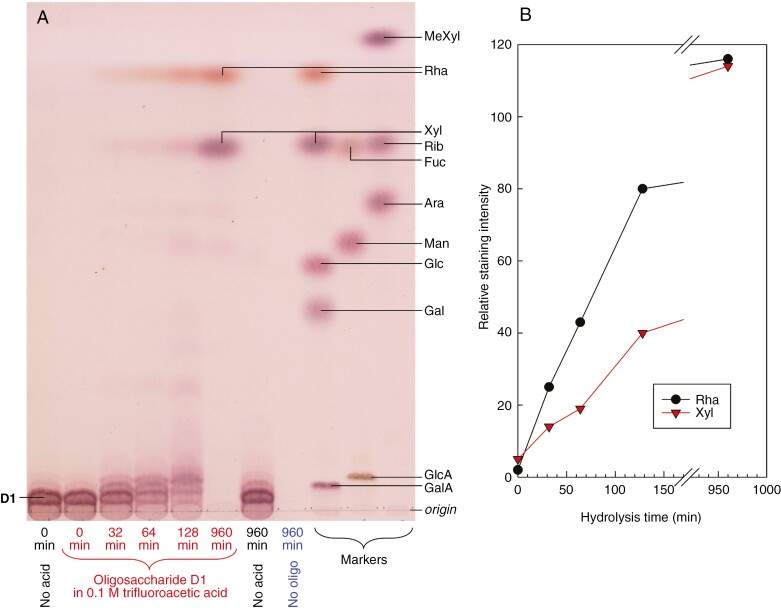
Mild acid lability of Driselase-released oligosaccharide D1. D1 (see Supplementary Data Fig. S2A) was submitted to mild acid hydrolysis in 0.1 M TFA at 80 °C for 0–960 min. Controls included incubation at 80 °C for 960 min in the absence of acid, and acid incubation in the absence of substrate. (A) Analysis of the products by TLC in EPAW with two ascents. (B) Staining intensity of the two major monosaccharides obtained, relative to the intensity of a Rha or Xyl marker (1.5 µg).

The largest oligosaccharide (D1) of this collection was tested for its susceptibility to graded acid hydrolysis: Rha was released sooner than Xyl (Fig. 7). This indicates a probable terminal position for the Rha residues, as side-chains of the oligomer. Between 32 and 128 min hydrolysis, a series of moderate-sized oligomers was visible. By 960 min, Rha and Xyl had been released in approximately equal masses, and the original oligomer and small intermediate oligosaccharides had been completely hydrolysed.

### *Acid hydrolysis of* Klebsormidium *pectic fraction*

**Fig. 8.**
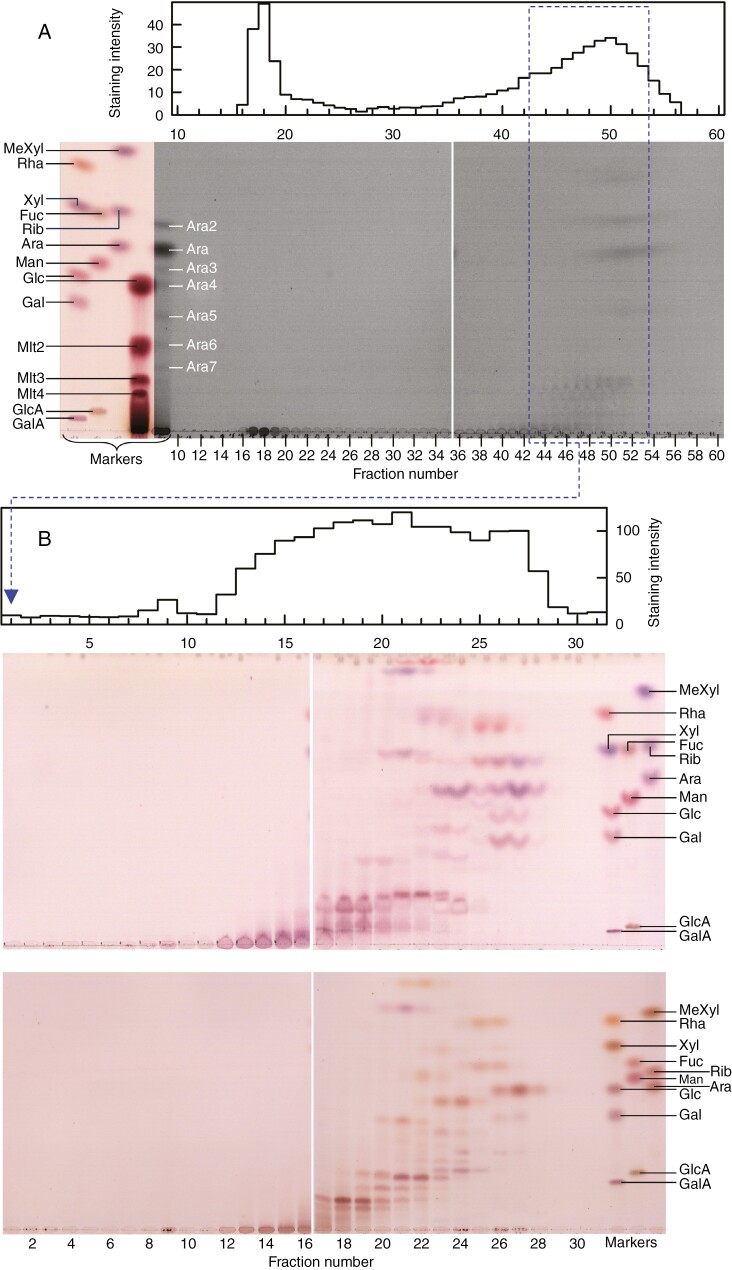
Gel-permeation chromatography of partly acid-hydrolysed *Klebsormidium* pectic fraction. Pectic fraction P2 was partly hydrolysed in 0.5 M TFA at 80 °C for 1 h. (A) The products were resolved on Bio-Gel P-30: above, profile of total carbohydrate revealed by thymol dot-blots; below, fractions 10–60 analysed individually by TLC in BAW. Fractions 43 to 53 (highlighted in blue) were pooled. (B) This pool was re-run on Bio-Gel P-2: top, total carbohydrate profile; middle, fractions 1–31 run individually by TLC in EPAW; bottom, ditto in BAW. Note: In both (A) and (B), plates were attached together digitally for clarity.

Mild acid hydrolysis of polysaccharides can generate oligomers that help in polymer analysis. Although acid hydrolysis is not as selective as enzymic hydrolysis, the varying susceptibilities to acid hydrolysis of certain glycosidic bonds allows for the differential release of particular oligomers. For example, furanose residues (such as Ara often is) are particularly acid labile, whereas uronic acid residues are far more acid resistant (reviewed by Fry, 2000). A range of conditions for the production of defined oligomers via mild acid hydrolysis was therefore tested on the *Klebsormidium* P2 pectic fraction (0.5–2.0 M TFA, 40–120 °C, 0.5–2 h). The most informative results came with 0.5 M TFA at 80 °C for 1 h, which gave two main peaks resolved by gel-permeation chromatography on Bio-Gel P-30 (fractionation range DP ~13–200; Fig. 8A): one around fraction 18 (DP too big to migrate on TLC), and a second, wide peak centred on fraction 50, which contained several oligomers of DP ~4 (judged by comparison with malto-oligosaccharide markers) around fraction 48 and the previously detected monomers (Rha, Xyl, Ara, Glc and Gal) around fraction 52.

**Fig. 9.**
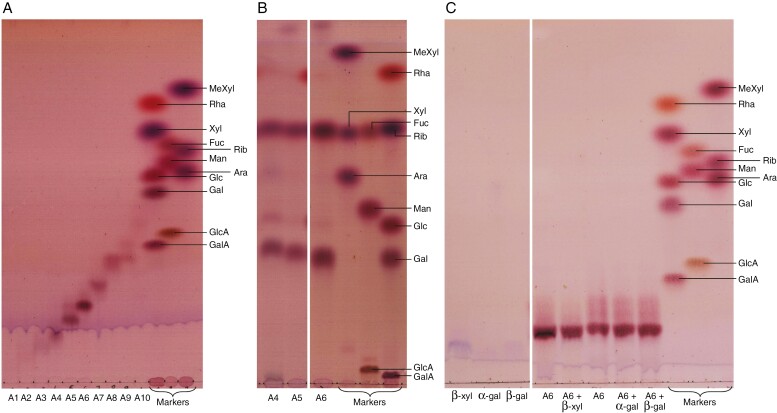
Thin-layer chromatography (TLC) of moderate-sized oligosaccharides from partly acid-hydrolysed *Klebsormidium* pectic fraction and their subsequent analysis. (A) Ten oligosaccharides (A1–A10) obtained by partial acid hydrolysis of Bio-Gel P-2 fraction 19 (see Fig. 8B) and purified further by preparative TLC (not shown) were developed in BAW with two ascents. (B) Oligosaccharides A4, A5 and (in a separate experiment) A6 were then completely acid hydrolysed, and the monosaccharides obtained were resolved by TLC in EPAW with two ascents. (C) Oligosaccharide A6 was incubated with or without β-d-xylosidase (β-xyl), α-d-galactosidase (α-gal) or β-d-galactosidase (β-gal) in the same conditions as in Fig. 5. Controls had the enzymes without substrate. The TLC plates were developed in BAW with two ascents. Note: two sections of the same plate were attached together digitally for clarity.

Fractions 43–53 from Bio-Gel P-30 (Fig. 8A) were pooled and re-chromatographed on Bio-Gel P-2. One broad peak of thymol-positive sugars was observed in the Bio-Gel P-2 fractions 12–28 (Fig. 8B). Many of these sugars were well resolved by TLC (Fig. 8B, middle and bottom). Nevertheless, to resolve overlapping sugars better, we re-ran fractions 17–27 by preparative TLC in EPAW: fractions 17–19 with three ascents and fractions 20–27 with two. Spots from these TLC plates (unstained) were eluted, affording ten mild-acid-released oligosaccharides (A1–A10). Portions of each of these were examined by analytical TLC in BAW (Fig. 9A). Additional portions of the three major bands (A4, A5 and A6) were fully acid hydrolysed and the products resolved by TLC in EPAW; each released mainly Xyl and Gal (ratio ~1:2; Fig. 9B), although A4 also released traces of Glc, Ara and Rha. Oligosaccharide A6, although composed predominantly of Gal and Xyl residues, was resistant to α- and β-d-galactosidase and β-d-xylosidase (Fig. 9C), hence no conclusive evidence for the anomerism could be obtained.

## DISCUSSION

In this work, the ‘pectic’ fraction from *Klebsormidium* cell walls was solubilized under a series of pH, buffer and temperature conditions, and conclusions were drawn about this novel polymer. Our use of the term ‘pectic’ rests on an operational definition (extractability in hot, slightly acidic buffers), regardless of the chemical composition of the polysaccharides. In fact, as we show here, the *Klebsormidium* pectic fraction differs fundamentally in its chemical composition from the classic pectins of the later-diverging charophytes and the land plants.

Pretreating the AIR with alkali led to higher and faster polysaccharide yields, whereas pretreatment with EPG or with α-amylase did not appreciably affect extraction. We presume that alkali treatment loosens interactions within the cell wall, leading to a quicker and more efficient release of polymers. In land-plant cell walls, commonly considered interactions between the different fractions (pectin, hemicelluloses and cellulose) are non-covalent bonds (hydrogen bonds, Van der Waals interactions and ionic bonds), esters [e.g. diferuloyl esters (Yu *et al.*, 2005)] or glycosidic linkages [e.g. between pectin and hemicelluloses (Thompson and Fry, 2000) and between pectin and cellulose (in carrot, but not in tomato and strawberry; Broxterman and Schols, 2018)]. Glycosidic bonds are unlikely to be cleaved by mild alkali, and ester cross-links are unlikely to be present in *Klebsormidium* cell walls because they contain no detectable carboxylic acids. Thus, non-covalent bonds are the most likely to be impacted by incubation in dilute alkali, suggesting that they are an important driver of cell-wall cohesion in *Klebsormidium*. The second phenomenon observed, i.e. the lack of impact of EPG and α-amylase on pectic fraction extraction yields, is an indication that homogalacturonan and starch might both be absent from the AIR.

These conclusions are supported by the impact of varying conditions on the extractability of pectic fractions (P1 and P2) from *Klebsormidium* (Fig. 1C, D). In land plants, pectin is more readily extractable with chelating agents (e.g. oxalate at pH ~4–5), because these disturb the egg-box structure of calcium-bound homogalacturonan chains (Walkinshaw and Arnott, 1981) in addition to providing optimal pH and temperature conditions (Fry, 2000). However, the *Klebsormidium* pectic fraction was most readily extractable with formate, which is non-chelating, and none of the *Klebsormidium* polysaccharide fractions contained detectable uronic acid residues, agreeing with previous reports (Popper and Fry, 2003; Sørensen *et al.*, 2011; O’Rourke *et al.*, 2015).

Taken together, this points to a radically different organization of the cell-wall matrix compared with the Phragmoplastophyta (*sensu* Hall *et al.*, 2020; i.e. later-diverging charophytes plus all land plants), which contain homogalacturonan as a major pectic domain (Albersheim *et al.*, 2011; Domozych *et al.*, 2014). Indeed, the main monomer residues detected in the *Klebsormidium* pectic fraction were Xyl, Gal, Rha and small amounts of Glc, Ara and Man. Of these, only Rha, Gal and Ara are common monomers in land-plant pectic domains; Xyl, Man and Glc are characteristic of land-plant hemicelluloses.

Enzymic digestion of the *Klebsormidium* pectic fraction showed that endo-α-(1→5)-l-arabinanase, endo-β-(1→4)-d-xylanase, endo-β-(1→4)-d-mannanase 2 (Table 1 and Fig. 4C) and α-l-galactosidase released mono- or oligosaccharides. In contrast, endo-β-(1→4)-d-galactanase, endo-β-(1→4)-d-mannanase 1, lichenase, β-d-xylosidase and β-d-galactosidase all gave negative results. This points to the presence of arabinan-like domains, hetero-β-xylans and α-Gal non-reducing termini. It also points to the absence of β-galactans, β-mannans and MLGs. The heteroxylans could be viewed as polymers halfway between hemicellulose and pectin; alkali helps to cleave non-covalent bonds between xylans and cellulose, helping these (nominally pectic fraction) polymers to be released.

On digestion with Driselase, a series of oligomers was released, predominantly composed of Rha and Xyl residues, and we conclude that Rha is likely to be a side-chain, in view of its greater susceptibility to release as a monosaccharide by mild acid. Given previous enzymic data suggesting the presence of heteroxylans [albeit mainly arabinoxylan (Jensen *et al,* 2018; Hsieh and Harris, 2019)], it is possible that a portion of the *Klebsormidium* pectic fraction is a rhamnoxylan: a backbone of (1→4)-β-linked Xyl residues substituted by Rha residues.

On mild acid hydrolysis of the *Klebsormidium* pectic fraction, another series of oligomers, rich in Gal and Xyl (but very little Rha), was released. These oligomers might indicate a distinct ‘pectic’ galactoxylan that is not susceptible to Driselase digestion and is partly resistant to mild acid hydrolysis.

Overall, this body of data suggests a pectic fraction with minor Ara-rich domains (evidenced by enzymic digestion), rhamnoxylan domains consisting of a Xyl-rich backbone with Rha side-chains (evidenced by oligomers produced by Driselase treatment), and galactoxylan domains with an as-yet undefined structure (evidenced by oligomers produced by mild acid hydrolysis). Some sugars detected in the total hydrolysate were not found in the oligomers, such as mannose and Glc. Their position in the ‘pectic’ polysaccharide structure remains unknown. The susceptibility of the *Klebsormidium* pectic fraction to Driselase should also be noted, because it indicates that sufficient glycosidic bonds are land-plant-like to produce a range of oligomers, showing that the *Klebsormidium* cell wall is not totally alien to the rest of the Streptophyta.

The unique pectic fraction structure here partly described is unusual for many reasons, the most obvious one being the absence of uronic acids. In the land-plant cell wall, homogalacturonan (with calcium bridges) forms a gel contributing to cell–cell adhesion and cell shaping. Other mechanisms must be in place to ensure such basic cell wall functions in *Klebsormidium*, creating approximately cylindrical cells joined end to end. Such mechanisms might be based on hydrophobic interactions, because such mechanisms have been observed between arabinan side-chains of land-plant pectin and cellulose (Zykwinska *et al*, 2007, 2008; Gawkowska *et al*, 2018). Hydrophobic interactions would radically change the nutritional requirements of the alga; although calcium ions are normally an important part of the cell-wall make-up, they would be redundant in *Klebsormidium* walls. This might be one of the factors contributing to the tolerance by *Klebsormidium* of acidic, metal-rich environments. This genus of algae is commonly found in acid mine drainages, which are often particularly rich in dissolved aluminium ions (Al^3+^) and particularly acidic. In such conditions, most plants suffer a rigidification of their cell walls (Zhu *et al.*, 2012) as Ca^2+^ ions are replaced by Al^3+^, which has a higher affinity for pectin, thus tightening the homogalacturonan network and hindering the correct mechanical functions of the cell wall. The absence of uronic acid residues, implying the absence of ionic bonds, frees the *Klebsormidium* cell wall from such constraints and might contribute to the abundance of these species in extreme environments.

The extractable pectin present in the Phragmoplastophyta is rich in homogalacturonan domains with varying degrees of methyl-esterification (Cherno *et al.*, 1976; Popper and Fry, 2003; Domozych *et al.*, 2007, 2009, 2010; Eder and Lütz-Meindl, 2008, 2010; Albersheim *et al.*, 2011; Sørensen *et al.*, 2011; O’Rourke *et al.*, 2015; Anderson, 2016). In contrast, we report here that the *Klebsormidium* pectic fraction lacks uronic acids. Of the charophytes that diverged earlier than the Klebsormidiophyceae, *Chlorokybus* possesses a ‘pseudopectin’ that is anionic (like in the Phragmoplastophyta but unlike in *Klebsormidium*) and comprises residues of neutral sugars (Ara ≈ Glc > l-Gal > Xyl, but negligible Rha and d-Gal; a unique combination), uronic acids (GlcA, GalA) and anionic sulphate groups (Rapin *et al.*, 2023). *Chlorokybus* pseudopectin is resistant to enzymes that attack land-plant pectin, especially endo-α-(1→4)-poly-d-galacturonase (indicating the absence of homogalacturonan), but also endo-α-(1→5)-l-arabinanase, endo-β-(1→4)-d-galactanase, endo-β-(1→4)-d-xylanase, α- and β-d-galactosidases and ‘Driselase’. Thus, despite containing GalA residues, *Chlorokybus* pseudopectin is clearly very different from Phragmoplastophyta pectin. The evolution of Phragmoplastophyta pectin from the polysaccharides of the earliest-diverging streptophytes was evidently a multifaceted process involving loss of sulphate, most l-Gal and most d-GlcA; reconfiguration of GalA, Ara and Xyl; and gain of Rha. The Klebsormidiophyceae diverged between the Chlorokybophyceae and the Phragmoplastophyta, and, as we report here, their pectic fraction is radically distinct from both the pseudopectin of the earlier-diverging *Chlorokybus* and the classic pectin of the later-diverging Phragmoplastophyta. Overall, it seems that during their evolution, the early streptophytes ‘experimented’ audaciously with the structure and function of their hot-water-extractable (‘pectic’, *sensu lato*) cell-wall polysaccharides. In particular, the evolution of classic pectin, based on calcium-bridged α-(1→4)-GalA residues, coincided with the evolution of the phragmoplast, whose function hinges on this type of pectin (Anderson, 2016).

Finally, we should re-emphasize that although the cell-wall fraction studied in this work is described as ‘pectin’ throughout, it does not share the most obvious structural features usually considered characteristic of pectin. We call it the pectic fraction because of its extractability properties. However, we currently lack a klebsormidiacean (and, potentially, chlorokybacean)-specific framework for describing algal cell walls. The question widens when we consider charophyte cell-wall characterization in general, because some of their polymers do not fit the usual criteria. Depending on the results of further characterization of the *Klebsormidium* cell wall, maybe its pectic fraction will be renamed ‘klebsormidiacean heteroxylan’; a more accurate, albeit longer name.

## SUPPLEMENTARY DATA

Supplementary data are available at *Annals of Botany* online and consist of the following.

Figure S1: sugar residue composition of the pectic fraction from *Klebsormidium* alcohol-insoluble residue. Figure S2: separation and sugar residue composition of medium-sized oligosaccharides obtained by Driselase digestion of *Klebsormidium* pectic fraction.

mcae154_suppl_Supplementary_Figure

## APPENDIX: ABBREVIATIONS

αA, AIR receiving α-amylase pretreatment

αC, α-cellulose

A, AIR receiving alkali pretreatment

A+E, AIR receiving alkali then endopolygalacturonase pretreatment

AIR, alcohol-insoluble residue

Ara, l-arabinose

Ara_2–8_, α-(1→5)-Ara di- to octasaccharides

BAW, butan-1-ol/acetic acid/water (4:1:1 by volume)

DP, degree of polymerization

E, AIR receiving endopolygalacturonase pretreatment

EPAW, ethyl acetate/pyridine/acetic acid/water (6:3:1:1 by volume)

EPG, endopolygalacturonase

Fuc, l-fucose

Gal, d-galactose

Gal_2–4_, β-(1→4)-Gal di- to tetrasaccharides

GalA, d-galacturonic acid

Glc, d-glucose

GlcA, d-glucuronic acid

Ha, hemicellulose a

Hb, hemicellulose b

Man, d-mannose

MeXyl, 2-*O*-methyl-d-xylose

MLG, mixed-linkage (1→3),(1→4)-β-d-glucan

Mlt_2–4_, maltose to maltotetraose

N, AIR receiving no pretreatment

P1, P2, successively extracted pectic fractions

Rha, l-rhamnose

Rib, d-ribose

TFA, trifluoroacetic acid

TLC, thin-layer chromatography

W, wash (buffer-extracted polysaccharides obtained after extraction of hemicelluloses)

Xyl, d-xylose

## References

[CIT0001] Albersheim P , Darvill A, Roberts K, Sederoff R, Staehelin A. 2011. Plant cell walls. New York: Garland Science Taylor and Francis Group.

[CIT0002] Anderson CT. 2016. We be jammin’: an update on pectin biosynthesis, trafficking and dynamics. *Journal of Experimental Botany* 67: 495–502. 26590862 10.1093/jxb/erv501

[CIT0003] Bischoff HW , Bold HC. 1963. Some soil algae from Enchanted Rock and related algal species. Phycological Studies IV. University of Texas Publications 6318: 1–95.

[CIT0004] Broxterman SE , Schols HA. 2018. Interactions between pectin and cellulose in primary plant cell walls. Carbohydrate Polymers 192: 263–272. 29691020 10.1016/j.carbpol.2018.03.070

[CIT0005] Cherno NK , Dudkin MS, Areshidze IV. 1976. Pectin substances of *Chara aculeolata*. Chemistry of Natural Compounds 12: 633–635.

[CIT0006] Cook ME , Graham LE. 2017. Chlorokybophyceae, Klebsormidiophyceae, Coleochaetophyceae. In: Archibald JM, Simpson AGB, Slamovits CH, eds. Handbook of the protists. Cham: Springer, 185–204.

[CIT0007] Cracraft J , Donoghue MJ. 2004. Assembling the tree of life. New York, NY; online edn, Oxford Academic: Oxford University Press.

[CIT0008] Domozych DS , Serfis A, Kiemle SN, Gretz MR. 2007. The structure and biochemistry of charophycean cell walls: I. Pectins of *Penium margaritaceum*. Protoplasma 230: 99–115. 17111095 10.1007/s00709-006-0197-8

[CIT0009] Domozych DS , Sørensen I, Willats WGT. 2009. The distribution of cell wall polymers during antheridium development and spermatogenesis in the charophycean green alga, *Chara corallina*. Annals of Botany 104: 1045–1056. 19696037 10.1093/aob/mcp193 PMC2766190

[CIT0010] Domozych DS , Sørensen I, Pettolino FA, Bacic A, Willats WGT. 2010. The cell wall polymers of the charophycean green alga *Chara corallina*: immunobinding and biochemical screening. International Journal of Plant Sciences 171: 345–361.

[CIT0011] Domozych DS , Sørensen I, Popper ZA, et al. 2014. Pectin metabolism and assembly in the cell wall of the charophyte green alga *Penium margaritaceum*. Plant Physiology 165: 105–118. 24652345 10.1104/pp.114.236257 PMC4012572

[CIT0012] Edelmann HG , Fry SC. 1992. Factors that affect the extraction of xyloglucan from the primary cell walls of suspension-cultured rose cells. Carbohydrate Research 228: 423–431.

[CIT0013] Eder M , Lütz-Meindl U. 2008. Pectin-like carbohydrates in the green alga *Micrasterias* characterized by cytochemical analysis and energy filtering TEM. Journal of Microscopy 231: 201–214. 18778418 10.1111/j.1365-2818.2008.02036.x

[CIT0014] Eder M , Lütz-Meindl U. 2010. Analyses and localization of pectin-like carbohydrates in cell wall and mucilage of the green alga *Netrium digitus*. Protoplasma 243: 25–38. 19340523 10.1007/s00709-009-0040-0 PMC2892062

[CIT0015] Elster J , Degma P, Kováčik L, Valentová L, Šramková K, Pereira AB. 2008. Freezing and desiccation injury resistance in the filamentous green alga *Klebsormidium* from the Antarctic, Arctic and Slovakia. Biologia 63: 843–851.

[CIT0016] Fry SC. 2000. The growing plant cell wall: chemical and metabolic analysis, Reprint edn. Caldwell, NJ: The Blackburn Press. ISBN 1-930665-08-3.

[CIT0017] Gawkowska D , Cybulska J, Zdunek A. 2018. Structure-related gelling of pectins and linking with other natural compounds: a review. Polymers 10: 762. 30960687 10.3390/polym10070762 PMC6404037

[CIT0018] Glass SE , McCourt RM, Gottschalk SD, Lewis LA, Karol KG. 2023. Chloroplast genome evolution and phylogeny of the early-diverging charophycean green algae with a focus on the Klebsormidiophyceae and *Streptofilum*. Journal of Phycology 59: 1133–1146. 37548118 10.1111/jpy.13359

[CIT0049] Hall JD , Lewis LA, McCourt RM, Delwiche CF, Mishler B, Karol, KG. 2020. Chlorophyta. In: de Queiroz K, Cantino PD, Gauthier JA, Eds. Phylonyms: A companion to the PhyloCode. Boca Raton: CRC Press, 183–186.

[CIT0019] Herburger K , Holzinger A. 2015. Localization and quantification of callose in the streptophyte green algae *Zygnema* and *Klebsormidium*: correlation with desiccation tolerance. Plant and Cell Physiology 56: 2259–2270. 26412780 10.1093/pcp/pcv139 PMC4650865

[CIT0020] Holzinger A , Pichrtová M. 2016. Abiotic stress tolerance of charophyte green algae: new challenges for omics techniques. Frontiers in Plant Science 7: 678. 27242877 10.3389/fpls.2016.00678 PMC4873514

[CIT0021] Hsieh YSY , Harris PJ. 2019. Xylans of red and green algae: what is known about their structures and how they are synthesised? Polymers 11: 354. 30960338 10.3390/polym11020354 PMC6419167

[CIT0022] Jarvis MC , Briggs SPH, Knox P. 2003. Intercellular adhesion and cell separation in plants. Plant, Cell and Environment 26: 977–989.

[CIT0023] Jensen JK , Busse-Wicher M, Poulsen CP, et al. 2018. Identification of an algal xylan synthase indicates that there is functional orthology between algal and plant cell wall biosynthesis. The New Phytologist 218: 1049–1060. 29460505 10.1111/nph.15050 PMC5902652

[CIT0024] Jork H , Funk W, Fischer W, Wimmer H. 1994. Thin layer chromatography: reagents and detection methods. Weinheim: WCH.

[CIT0025] Kaplan F , Lewis LA, Wastian J, Holzinger A. 2012. Plasmolysis effects and osmotic potential of two phylogenetically distinct alpine strains of *Klebsormidium* (Streptophyta). Protoplasma 249: 789–804. 21979310 10.1007/s00709-011-0324-z

[CIT0026] Karsten U , Herburger K, Holzinger A. 2016. Living in biological soil crust communities of African deserts—physiological traits of green algal *Klebsormidium* species (Streptophyta) to cope with desiccation, light and temperature gradients. Journal of Plant Physiology 194: 2–12. 26422081 10.1016/j.jplph.2015.09.002 PMC4710676

[CIT0027] Karsten U , Holzinger A. 2014. Green algae in alpine biological soil crust communities: acclimation strategies against ultraviolet radiation and dehydration. Biodiversity and Conservation 23: 1845–1858. 24954980 10.1007/s10531-014-0653-2 PMC4058318

[CIT0028] Karsten U , Lütz C, Holzinger A. 2010. Ecophysiological performance of the aeroterrestrial green alga *Klebsormidium crenulatum* (Charophyceae, Streptophyta) isolated from an alpine soil crust with an emphasis on desiccation stress. Journal of Phycology 46: 1187–1197. 10.1111/j.1529-8817.2011.00980.x 27021989

[CIT0029] Karsten U , Pröschold T, Mikhailyuk T, Holzinger A. 2013. Photosynthetic performance of different genotypes of the green alga *Klebsormidium* sp. (Streptophyta) isolated from biological soil crusts of the Alps. Algological Studies 142: 45–62.

[CIT0030] Kitzing C , Pröschold T, Karsten U. 2014. UV-induced effects on growth, photosynthetic performance and sunscreen contents in different populations of the green alga *Klebsormidium fluitans* (Streptophyta) from alpine soil crusts. Microbial Ecology 67: 327–340. 24233286 10.1007/s00248-013-0317-x

[CIT0031] Margolles A , de los Reyes-Gavilán CG. 2003. Purification and functional characterization of a novel α-l-arabinofuranosidase from *Bifidobacterium longum* B667. Applied and Environmental Microbiology 69: 5096–5103. 12957891 10.1128/AEM.69.9.5096-5103.2003 PMC194971

[CIT0032] Míguez F , Holzinger A, Fernandez-Marin B, García-Plazaola JI, Karsten U, Gustavs L. 2020. Ecophysiological changes and spore formation: two strategies in response to low-temperature and high-light stress in *Klebsormidium* cf. *flaccidum* (Klebsormidiophyceae, Streptophyta). Journal of Phycology 56: 649–661. 31957017 10.1111/jpy.12971 PMC7612455

[CIT0033] Mikkelsen MD , Harholt J, Westereng B, et al. 2021. Ancient origin of fucosylated xyloglucan in charophycean green algae. Communications Biology 4: 754. 34140625 10.1038/s42003-021-02277-w PMC8211770

[CIT0034] Nagao M , Matsui K, Uemura M. 2008. *Klebsormidium flaccidum*, a charophycean green alga, exhibits cold acclimation that is closely associated with compatible solute accumulation and ultrastructural changes. Plant, Cell and Environment 31: 872–885. 10.1111/j.1365-3040.2008.01804.x 18315534

[CIT0050] Nguyen-Phan TC , Fry SC. 2019. Functional and chemical characterization of XAF: a heat-stable plant polymer that activates xyloglucan endotransglucosylase/hydrolase (XTH). Annals of Botany 124: 131–147. 31147677 10.1093/aob/mcz050 PMC6676392

[CIT0035] O’Rourke C , Gregson T, Murray L, Sadler IH, Fry SC. 2015. Sugar composition of the pectic polysaccharides of charophytes, the closest algal relatives of land-plants: presence of 3-*O*-methyl-d-galactose residues. Annals of Botany 116: 225–236. 26113633 10.1093/aob/mcv089 PMC4512192

[CIT0036] Popper ZA , Fry SC. 2003. Primary cell wall composition of bryophytes and charophytes. Annals of Botany 91: 1–12. 12495914 10.1093/aob/mcg013 PMC4240358

[CIT0037] R Core Team. 2021. R: a language and environment for statistical computing. Vienna, Austria: R Foundation for Statistical Computing. https://www.r-project.org/(6 May 2024, date last accessed).

[CIT0038] Rapin MN , Murray L, Sadler IH, Bothwell JH, Fry SC. 2023. Same but different — pseudo-pectin in the charophytic alga *Chlorokybus atmophyticus*. Physiologia Plantarum 175: e14079. 38148229 10.1111/ppl.14079 PMC10953000

[CIT0039] Rippin M , Borchhardt N, Karsten U, Becker B. 2019. Cold acclimation improves the desiccation stress resilience of polar strains of *Klebsormidium* (Streptophyta). Frontiers in Microbiology 10: 1730. 31447802 10.3389/fmicb.2019.01730 PMC6691101

[CIT0040] Škaloud P , Lukešová A, Malavasi V, Ryšánek D, Hrčková K, Rindi F. 2014. Molecular evidence for the polyphyletic origin of low pH adaptation in the genus *Klebsormidium* (Klebsormidiophyceae, Streptophyta). Plant Ecology and Evolution 147: 333–345.

[CIT0041] Sørensen I , Pettolino FA, Bacic A, et al. 2011. The charophycean green algae provide insights into the early origins of plant cell walls. The Plant Journal 68: 201–211. 21707800 10.1111/j.1365-313X.2011.04686.x

[CIT0042] Thompson JE , Fry SC. 2000. Evidence for covalent linkage between xyloglucan and acidic pectins in suspension-cultured rose cells. Planta 211: 275–286. 10945222 10.1007/s004250000287

[CIT0043] Walkinshaw MD , Arnott S. 1981. Conformations and interactions of pectins: II. Models for junction zones in pectinic acid and calcium pectate gels. Journal of Molecular Biology 153: 1075–1085. 7343680 10.1016/0022-2836(81)90468-x

[CIT0044] Wang D , Kanyuka K, Papp-Rupar M. 2023. Pectin: a critical component in cell-wall-mediated immunity. Trends in Plant Science 28: 10–13. 36272890 10.1016/j.tplants.2022.09.003

[CIT0045] Yu P , McKinnon JJ, Christensen DA. 2005. Hydroxycinnamic acids and ferulic acid esterase in relation to biodegradation of complex plant cell walls. Canadian Journal of Animal Science 85: 255–267.

[CIT0046] Zhu XF , Shi YZ, Lei GJ, et al. 2012. *XTH31*, encoding an *in-vitro* XEH/XET-active enzyme, controls Al sensitivity by modulating *in-vivo* XET action, cell wall xyloglucan content and Al binding capacity in *Arabidopsis*. The Plant Cell 24: 4731–4747. 23204407 10.1105/tpc.112.106039 PMC3531863

[CIT0047] Zykwinska A , Thibault J-F, Ralet M-C. 2007. Organization of pectic arabinan and galactan side chains in association with cellulose microfibrils in primary cell walls and related models envisaged. Journal of Experimental Botany 58: 1795–1802. 17383990 10.1093/jxb/erm037

[CIT0048] Zykwinska A , Thibault J-F, Ralet M-C. 2008. Competitive binding of pectin and xyloglucan with primary cell wall cellulose. Carbohydrate Polymers 74: 957–961.

