# Supplementary material for: Pectin-like heteroxylans in the early-diverging charophyte Klebsormidium fluitans
Source: Ann Bot. 2024 Sep 21;134(7):1191–206. doi: 10.1093/aob/mcae154 (PMC11688530; doi:10.1093/aob/mcae154)
Supplement: mcae154_suppl_Supplementary_Figure [file mcae154_suppl_supplementary_figure.pptx]

## Slide 1
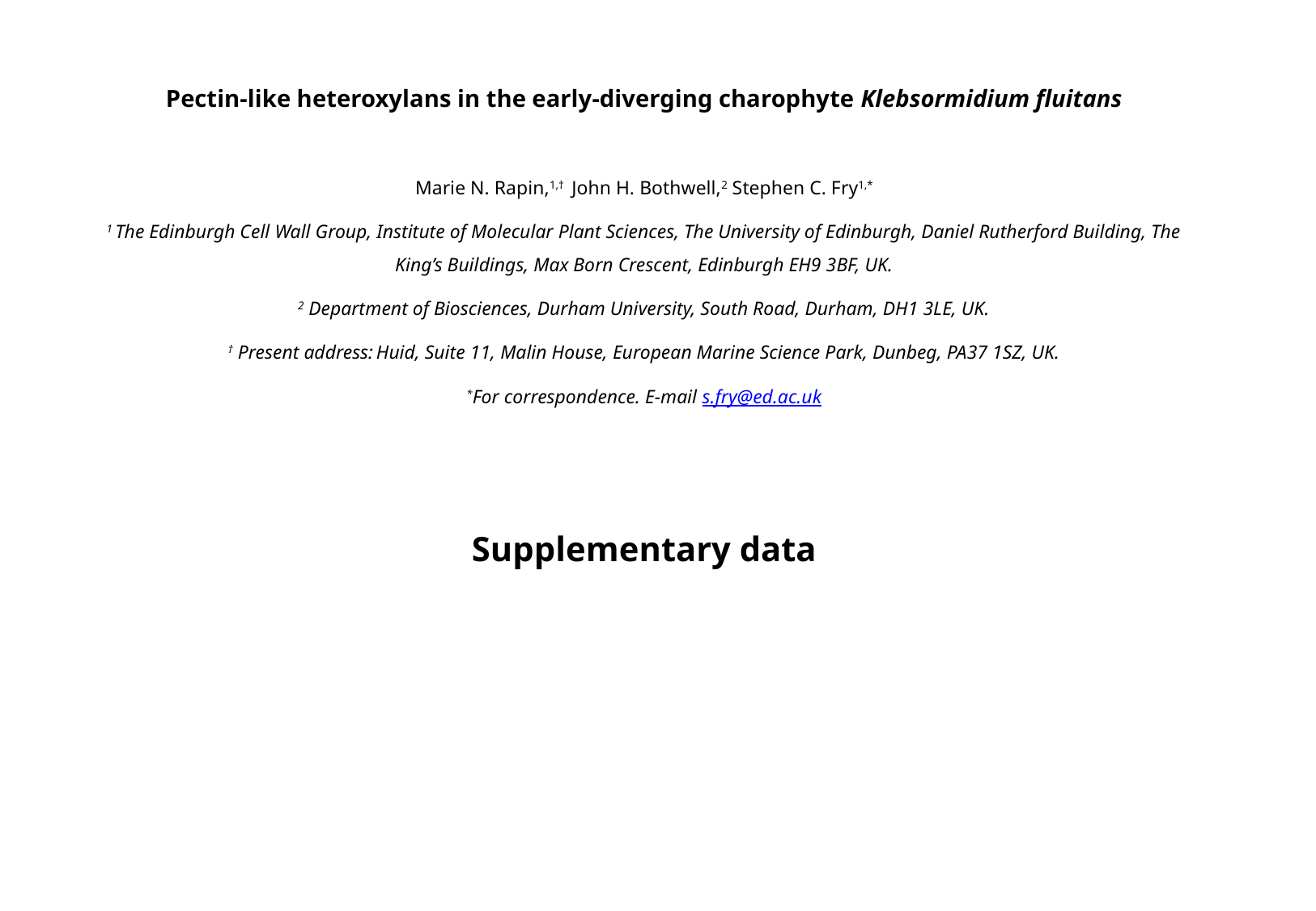

Pectin-like heteroxylans in the early-diverging charophyte Klebsormidium fluitans
Marie N. Rapin,1,† John H. Bothwell,2 Stephen C. Fry1,*
1 The Edinburgh Cell Wall Group, Institute of Molecular Plant Sciences, The University of Edinburgh, Daniel Rutherford Building, The King’s Buildings, Max Born Crescent, Edinburgh EH9 3BF, UK.
2 Department of Biosciences, Durham University, South Road, Durham, DH1 3LE, UK.
† Present address: Huid, Suite 11, Malin House, European Marine Science Park, Dunbeg, PA37 1SZ, UK.
*For correspondence. E-mail s.fry@ed.ac.uk
Supplementary data

## Slide 2
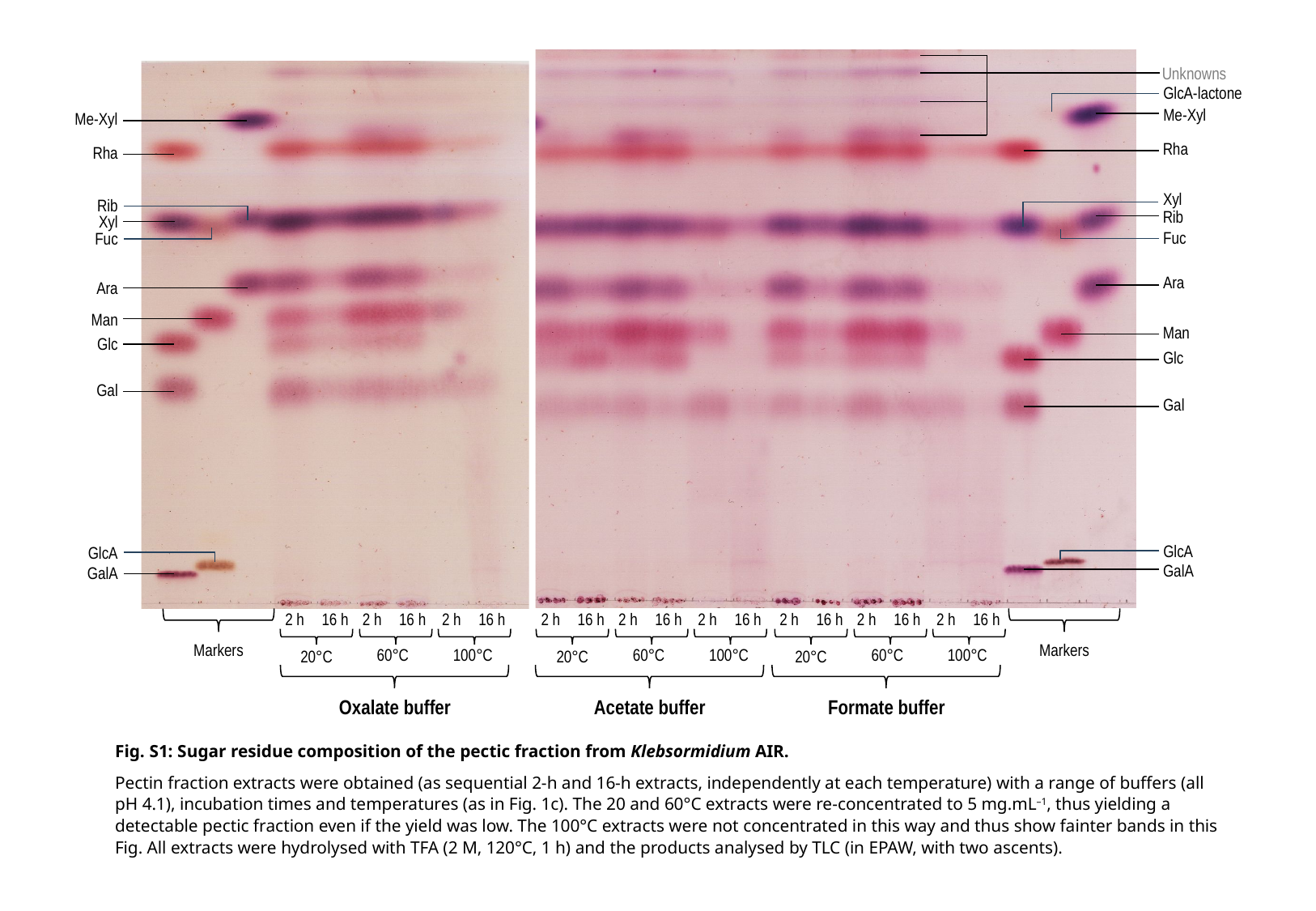

Me-Xyl
Me-Xyl
Rha
Rha
Xyl
Rib
Rib
Xyl
Fuc
Fuc
Ara
Ara
Man
Man
Glc
Glc
Gal
Gal
GlcA
GlcA
GalA
GalA
2 h
16 h
2 h
16 h
2 h
16 h
2 h
16 h
2 h
16 h
2 h
16 h
2 h
16 h
2 h
16 h
2 h
16 h
Markers
Markers
60°C
100°C
60°C
100°C
60°C
100°C
20°C
20°C
20°C
Oxalate buffer
Acetate buffer
Formate buffer
Unknowns
GlcA-lactone
Fig. S1: Sugar residue composition of the pectic fraction from Klebsormidium AIR.
Pectin fraction extracts were obtained (as sequential 2-h and 16-h extracts, independently at each temperature) with a range of buffers (all pH 4.1), incubation times and temperatures (as in Fig. 1c). The 20 and 60°C extracts were re-concentrated to 5 mg.mL–1, thus yielding a detectable pectic fraction even if the yield was low. The 100°C extracts were not concentrated in this way and thus show fainter bands in this Fig. All extracts were hydrolysed with TFA (2 M, 120°C, 1 h) and the products analysed by TLC (in EPAW, with two ascents).

## Slide 3
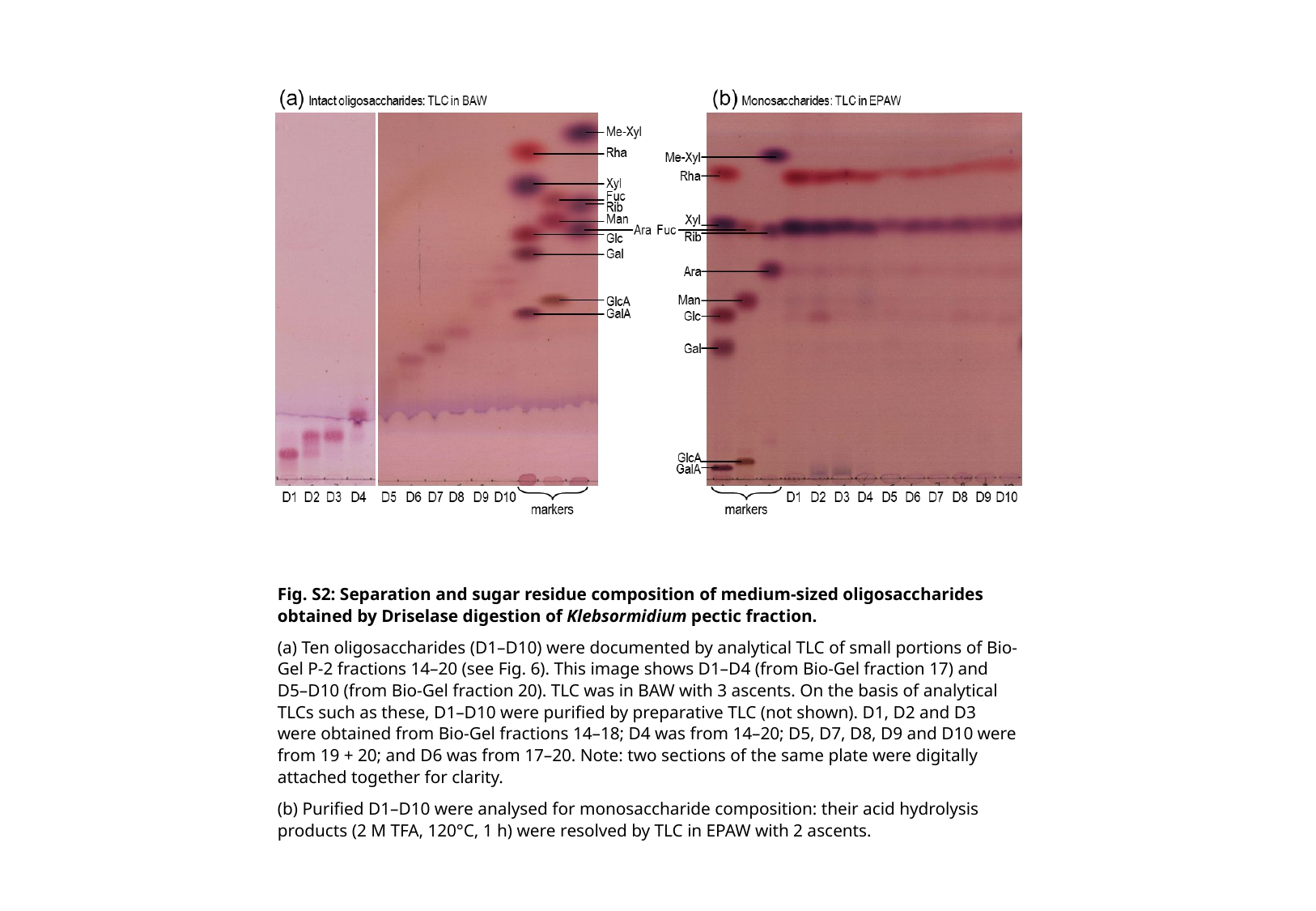

Fig. S2: Separation and sugar residue composition of medium-sized oligosaccharides obtained by Driselase digestion of Klebsormidium pectic fraction.
(a) Ten oligosaccharides (D1–D10) were documented by analytical TLC of small portions of Bio-Gel P-2 fractions 14–20 (see Fig. 6). This image shows D1–D4 (from Bio-Gel fraction 17) and D5–D10 (from Bio-Gel fraction 20). TLC was in BAW with 3 ascents. On the basis of analytical TLCs such as these, D1–D10 were purified by preparative TLC (not shown). D1, D2 and D3 were obtained from Bio-Gel fractions 14–18; D4 was from 14–20; D5, D7, D8, D9 and D10 were from 19 + 20; and D6 was from 17–20. Note: two sections of the same plate were digitally attached together for clarity.
(b) Purified D1–D10 were analysed for monosaccharide composition: their acid hydrolysis products (2 M TFA, 120°C, 1 h) were resolved by TLC in EPAW with 2 ascents.
